# Influence of Esophageal Endoscopic Submucosal Dissection on the Changes of Energy Metabolism during the Perioperative Period

**DOI:** 10.3390/cancers14082015

**Published:** 2022-04-15

**Authors:** Sae Kudo, Daisuke Chinda, Tadashi Shimoyama, Kohei Yasuda, Kazuki Akitaya, Tetsu Arai, Kuniaki Miyazawa, Shiro Hayamizu, Miyuki Yanagimachi, Toshiaki Tsukamoto, Masatoshi Kaizuka, Yohei Sawada, Tetsuya Tatsuta, Keisuke Hasui, Hidezumi Kikuchi, Hiroto Hiraga, Hirotake Sakuraba, Tatsuya Mikami, Shinsaku Fukuda

**Affiliations:** 1 Department of Gastroenterology and Hematology, Hirosaki University Graduate School of Medicine, Hirosaki 036-8562, Japan; ksae@hirosaki-u.ac.jp (S.K.); yasuda-k@hirosaki-u.ac.jp (K.Y.); kazukiakitaya0926@yahoo.co.jp (K.A.); teddyscello@icloud.com (T.A.); symboli1016@yahoo.co.jp (K.M.); hayaminn3@yahoo.co.jp (S.H.); m.kaizuka@hirosaki-u.ac.jp (M.K.); sawada-y@hirosaki-u.ac.jp (Y.S.); tatsuta.t@hirosaki-u.ac.jp (T.T.); hasui.k@hirosaki-u.ac.jp (K.H.); hhiraga@hirosaki-u.ac.jp (H.H.); hirotake@hirosaki-u.ac.jp (H.S.); sfukuda@hirosaki-u.ac.jp (S.F.); 2 Division of Endoscopy, Hirosaki University Hospital, Hirosaki 036-8563, Japan; 3 Aomori General Health Examination Center, Aomori 030-0962, Japan; tsimo@hirosaki-u.ac.jp; 4 Department of Endocrinology and Metabolism, Hirosaki University Graduate School of Medicine, Hirosaki 036-8562, Japan; yanagi@hirosaki-u.ac.jp; 5 Division of Rehabilitation, Hirosaki University Hospital, Hirosaki 036-8563, Japan; tukamoto@hirosaki-u.ac.jp; 6 Department of Community Medicine, Hirosaki University Graduate School of Medicine, Hirosaki 036-8562, Japan; hidezumi@hirosaki-u.ac.jp; 7 Center of Healthy Aging Innovation, Hirosaki University Graduate School of Medicine, Hirosaki 036-8562, Japan; tmika@hirosaki-u.ac.jp

**Keywords:** esophageal cancer, endoscopic submucosal dissection, energy metabolism, resting energy expenditure, indirect calorimeter

## Abstract

**Simple Summary:**

This study aimed to measure resting energy expenditure (REE) and assess the physical invasiveness of esophageal endoscopic submucosal dissection (ESD) during perioperative periods. In addition, the factors affecting changes in REE were investigated. Subjects were examined using an indirect calorimeter and the stress factor was calculated based on basal energy expenditure and body weight. REE/body weight on the day following ESD was significantly higher than that of the same day. The stress factor on the day after ESD was 1.11. The increase in WBC, neutrophil, and CRP levels was associated with the change in REE ratio. Among the factors affecting changes in energy metabolism, only the total resection area was associated with changes in REE. It is suggested that patients who undergo esophageal ESD require more attention in perioperative management when the resection area of the lesions is larger.

**Abstract:**

Esophageal endoscopic submucosal dissection (ESD) is considered to be more complex than gastric ESD. This study aimed to assess the physical invasiveness of esophageal ESD during perioperative periods by measuring resting energy expenditure (REE). The factors affecting REE that could be used to identify patients requiring perioperative management were also investigated. Overall, 75 patients who had undergone esophageal ESD were prospectively enrolled. REE, body weight, and basal energy expenditure were measured on the day of and the day following ESD. The mean REE/body weight was 20.2 kcal/kg/day on the day of ESD and significantly increased to 23.0 kcal/kg/day one day after ESD. The stress factor on the day after ESD was 1.11. White blood cell, neutrophil, and C-reactive protein levels increased on the day after ESD and correlated with the changes in REE. Among the factors including age, body mass index, total resection area, operation time, and sarcopenia, only the total resection area was associated with changes in REE. In conclusion, energy metabolism increases during the perioperative period for esophageal ESD. The increase in the stress factor for esophageal ESD was higher than that in gastric and colorectal ESD. Furthermore, patients with large resection areas require greater attention in perioperative management.

## 1. Introduction

Esophageal cancer is the sixth leading cause of cancer-related deaths worldwide, and its treatment depends on the activities of daily living and patient disease stage [1]. In older patients, full esophagectomy is considered a highly invasive procedure because treatment can deteriorate patient conditions [2]. Chemoradiotherapy (CRT) has been recognized as a reliable treatment regardless of disease stages in esophageal cancer. However, late toxicities caused by CRT often become fatal [2,3]. Endoscopic submucosal dissection (ESD) for superficial esophageal cancer is an efficacious treatment in terms of functional preservation and safety, especially in older patients [4,5,6,7]. ESD has been the standard treatment for superficial esophageal cancer in Japan since 2012 as it allows en bloc resection and has a low risk of local cancer recurrence [7,8]. Esophageal ESD is also recommended as a first-line treatment for clinical T1a-Epithelium/lamina propria mucosae diagnosed as noncircumferential esophageal cancer or in long-axis whole-circumferential esophageal cancers < 50 mm [7]. In addition, clinical T1a-muscularis mucosae/T1b-submucosa 1 esophageal cancer is also considered an indication for ESD if it is noncircumferential [7].

During operations such as ESD, hypermetabolism can be induced by inflammation and protein catabolism [9,10,11], reflecting the invasiveness of the surgery [10]. Differences in the degree of energy metabolism are caused by the production of pro-inflammatory cytokines and increased glucose oxidation during pathological stress [12]. In our previous study, changes in resting energy expenditure (REE) were measured using an indirect calorimeter during the perioperative period for gastric and colorectal ESD. Based on these results, the increase in REE on the day following ESD was low compared to that experienced after open surgery; therefore, ESD was recognized as a less invasive treatment [13,14]. 

However, esophageal ESD is considered a more complex procedure than gastric ESD [15,16]. In addition, bacteremia and postoperative stricture are perioperative complications common in esophageal ESD. Several studies have reported that blood cultures obtained 10 min after gastric ESD and 5 min after colorectal ESD were positive in 4.3% and 2.5% of patients, respectively [17,18]. Furthermore, the rate of bacteremia was 12–22% after esophageal bougienage and 0–52% after variceal sclerotherapy [17,19]. In these procedures, the cultured microorganisms were oral commensal bacteria [17,20]. Although a few studies have investigated bacteremia in esophageal ESD, bacteremia can occur after esophageal ESD. Postoperative stricture occurred in 90% of patients with lesions > 3/4 of the circumference of the lumen [21]. For the above reasons, it is possible that the physical invasiveness of esophageal ESD during the perioperative period differs from that of gastric and colorectal ESD. 

This study aimed to measure REE using an indirect calorimeter and assess physical invasiveness during the perioperative period for esophageal ESD. Additionally, we investigated the factors affecting changes in REE to identify cases that required additional care in perioperative management. We found that for esophageal ESD, energy metabolism increased during the perioperative period, and its degree of invasiveness was low compared with that of open surgery.

## 2. Materials and Methods

### 2.1. Measuring REE Using an Indirect Calorimeter

**Figure 1:**
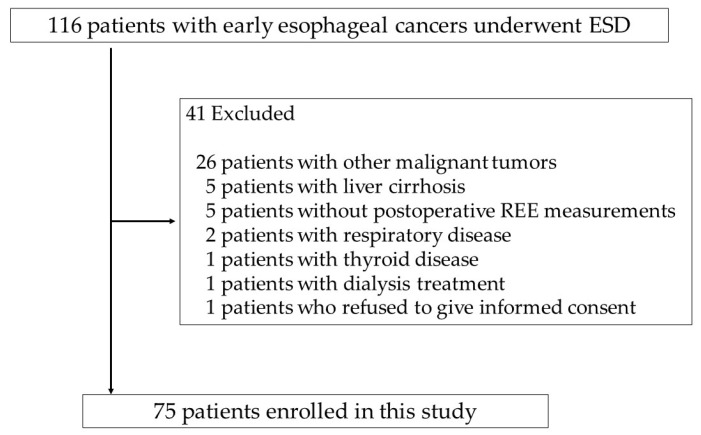
Flow chart of study patient selection.

Table 1 shows the patient characteristics. Between July 2013 and March 2019, we enrolled 116 consecutive patients who were to undergo esophageal ESD at Hirosaki University Hospital. We excluded patients with a history of liver cirrhosis, respiratory diseases, and thyroid diseases; those undergoing artificial dialysis; and those with other malignancies. Finally, 75 patients (median age 66 years; 67 men) were included in the study (Figure 1). We evaluated our sample using a power of 80% and a two-sided alpha level of 0.05. The standard deviation was computed using the prediction value based on data from our previous research on ESD for gastric cancer. As a result, the sample size of 75 had a statistical power of 0.9986 (REE/body weight) and a stress factor of 0.9995.

All ESD procedures (75 subjects) were carried out by five endoscopists certified by the Japan Gastroenterological Endoscopy Society (JGE) using a conventional single-channel video endoscope (GIF-Q260J, H260, or H290; Olympus, Tokyo, Japan) with a hood. The ESD procedure was performed using a water jet short needle knife (Flush Knife BT-S; DK2620J, Fujinon, Tokyo, Japan), a water jet hook knife (KD-620LR, Olympus, Tokyo, Japan), and a high-frequency generator with an automatically controlled system (VIO3 or VIO300D; ERBE, Tübingen, Germany). All patients were administered intravenous pethidine hydrochloride 25 mg/body and diazepam 5 mg/body or midazolam 2 mg/body prior to the start of ESD. These drugs were increased as appropriate depending on the degree of sedation, and a total of 25–100 mg/body pethidine hydrochloride and 5–20 mg/body diazepam or midazolam in 5–10 mg/body were finally administered before and during ESD. In addition to these drugs, dexmedetomidine hydrochloride was used for sedation in three patients. Dexmedetomidine hydrochloride was started at 6 μg/h/kg for the first 10 min and subsequently decreased to 0.4 μg/h/kg by continuous intravenous infusion.

REE was examined using an indirect calorimeter (METAVINE-N VMB-002N; VINE, Tokyo, Japan) on the day of and day after ESD [14,22]. Each patient fasted for more than 12 h, and REE was measured after 30 min of bed rest early in the morning on the day of and the day after ESD. The REE was determined three times and the average value was calculated. If the variability exceeded the range of 100 kcal, a fourth measurement was taken and REE was calculated using the average of the three values, excluding the one farthest from the average of the two medians. In a previous study, gas infusion tests in three indirect calorimeters showed great reproducibility and accuracy (within 3%) for energy expenditure [23]. In this study, three REE measurements were within the range of 100 kcal for 61 of 75 subjects (81.3%) before ESD and 62 of 75 subjects (82.6%) on postoperative day 1 (POD1), indicating high reproducibility. Afterward, ESD was performed and REE in the fasted state was similarly measured in the morning on POD1. Furthermore, the body weight of each patient was measured on the day of and the day after ESD. We used these measurements to calculate the changes in REE/body weight ratio.

### 2.2. BEE and Stress Factors

Basal energy expenditure (BEE) was assessed using the Harris–Benedict equation [24] based on Long’s method [25]. The REE was calculated by multiplying the BEE with stress and activity factors. The stress factor is one of the markers of hypermetabolic status [26,27]. Assuming REE/BEE on the day of ESD to be 1.00, the REE/BEE on POD1 can likely be considered a stress factor because the activity factors on the day of ESD and POD1 are the same in the resting state.

### 2.3. Hematological Response in Perioperative Period

The blood samples were collected in the morning on the day of ESD and POD1 after fasting for 12 h while resting. The number and differential counts of white blood cells (WBC) were measured using XE-5000 (Sysmex, Kobe, Japan). The serum levels of C-reactive protein (CRP) were measured using a JCA-BM6070 (EOL Ltd., Tokyo, Japan).

The change ratios of WBC, neutrophil, and CRP were investigated and correlated with changes in REE using the Spearman rank correlation coefficient.

### 2.4. Factor Associated with Change Ratio of REE during Perioperative Period

Age, body mass index (BMI), total resection area, operation time, and sarcopenia were evaluated as factors affecting changes in energy metabolism. The patients were divided into two groups. The cut-off values were as follows: age, 65 years (definition of older age provided by the World Health Organization [28]) and BMI, 25 (kg/m^2^) (definition of obesity [29]). The resection area was calculated by approximating an elliptical shape with the long and short axes of the resected specimens. For the nine subjects with multiple lesions, the total resection area was computed from all resected specimens. The total resection area and operation time were selected as the cut-offs for the median value (6.9 cm^2^ and 75 min). Sarcopenia is defined as a condition with low skeletal muscle mass and strength [30]. Skeletal muscle mass was expressed as skeletal muscle index (SMI) [31,32]. SMI evaluation using dual-energy X-ray absorptiometry or bioelectrical impedance analysis is the gold standard method; however, these methods are not easily available in common facilities [33]. As SMI correlates with psoas muscle index (PMI) [33,34], we used computed tomography in the preoperative period and estimated PMI by the cross-sectional area of the muscle at the third lumbar vertebra level normalized based on the patient’s height (cm^2^/m^2^) [35,36]. The cut-off value of PMI was defined as 6.0 cm^2^/m^2^ for men and 3.4 cm^2^/m^2^ for women [35]. These factors were computed between the two groups to determine the change ratio of REE from the preoperative to the postoperative state.

### 2.5. Statistical Analysis

The statistical analysis of the clinical data was performed using SPSS (version 24.0; SPSS Inc., Chicago, IL, USA) and R (R Foundation for Statistical Computing, version R-3.4.3). Data are expressed as medians and interquartile ranges. Statistical differences were analyzed using the paired *t*-test, the Wilcoxon signed-rank test, and the Mann–Whitney U test. We also used a non-parametric mixed regression model to examine the relationship between each factor and changes in REE, and performed multivariate analysis using generalized linear models. A *p*-value of less than 0.05 was considered statistically significant.

## 3. Results

### 3.1. REE, REE/Body Weight, and REE/BEE

The changes in REE, REE/body weight, and REE/BEE are shown in Table 2. The REE on POD1 was elevated in 56 of 75 patients (74.7%) compared with that on the day of ESD. The median of REE was 1194.7 kcal/day on the day of ESD, but 1340.0 kcal/day on POD1, significantly higher by 12.2% (*p* < 0.001).

There was no significant difference in REE changes between the five operators (data not shown).

**Figure 2:**
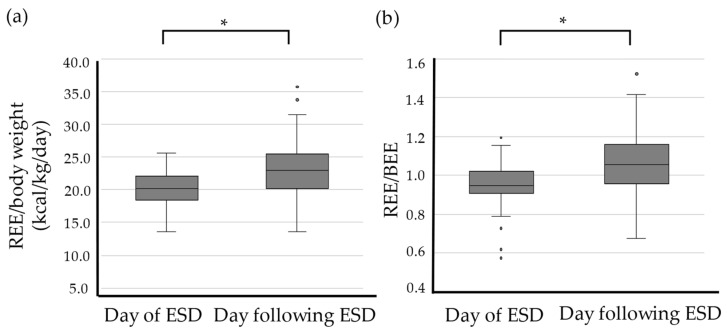
The changes of REE/body weight (**a**) and REE/BEE (**b**). Data are expressed as median (range). * *p <* 0.05: compared with the value on the day of ESD. ESD: endoscopic submucosal dissection; REE: resting energy expenditure; BEE: basal energy expenditure.

The REE/body weight ratio was elevated in 63 of the 75 patients (84.0%). The median REE/body weight was 20.2 kcal/kg/day on the day of ESD and significantly increased to 23.0 kcal/kg/day on POD1, significantly higher by 14.8% (*p <* 0.05; Figure 2a).

The median REE/BEE ratio was elevated in 64 of 76 patients (84.2%). There was a significant increase from 0.95 to 1.06 between the preoperative and postoperative statuses, respectively (*p <* 0.05; Figure 2b). The stress factor on POD1 was 1.11.

### 3.2. Correlation between Change Ratio of REE and Laboratory Findings

**Figure 3:**
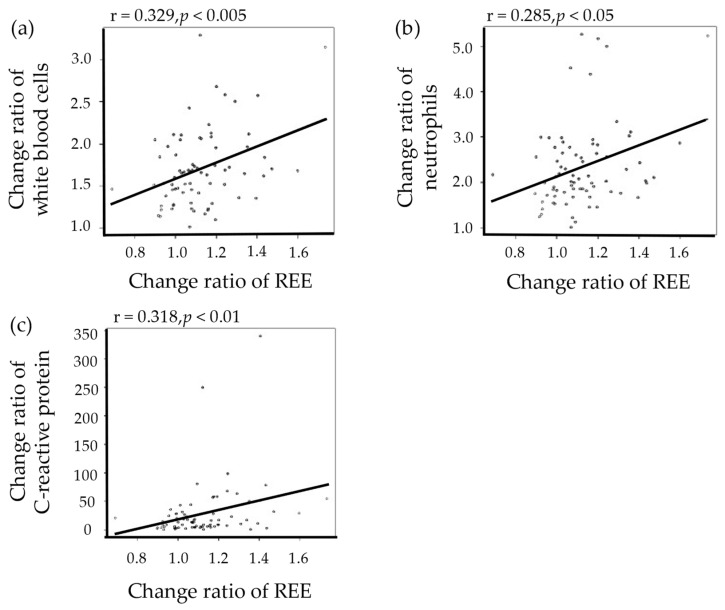
Correlations between the change ratios of REE and laboratory findings. (**a**) White blood cells. (**b**) Neutrophils. (**c**) C-reactive protein. REE: resting energy expenditure.

Table 3 shows the results of the changes in WBC (neutrophils, monocytes, eosinophils, basophils, and lymphocytes) and CRP. WBC, neutrophils, monocytes, and CRP increased significantly on POD1 in comparison with the day of ESD (*p* < 0.001). Conversely, eosinophils, basophils, and lymphocytes decreased significantly on the day after ESD (*p <* 0.001). The change ratios of laboratory findings (WBC, neutrophils, and CRP) showed significant positive correlations with the change ratio of REE during the perioperative period (r = 0.329, *p <* 0.005; r = 0.285 and *p <* 0.05, r = 0.318 and *p <* 0.01, respectively; Figure 3).

### 3.3. Factors Affecting Changes in REE

Table 4 shows the results of univariate analysis. The change ratio of REE in the small resection area (<6.9 cm^2^) group was significantly lower than that in the large resection area (≥6.9 cm^2^) group (*p* < 0.05). The size of the resection area was positively associated with the change ratio of REE. In contrast, there were no significant differences in age (<65 vs. ≥65), BMI (<25 kg/m^2^ vs. ≥25 kg/m^2^), operation time (<75 min vs. ≥75 min), or sarcopenia (PMI < 6.0 cm^2^/m^2^ vs. ≥6.0 cm^2^/m^2^ for men and <3.4 cm^2^/m^2^ vs. ≥3.4 cm^2^/m^2^ for women, respectively). Table 5 shows the results of the multivariate analysis using generalized linear models. There was no significant difference for each factor, but the value of estimates was the highest in the total resection area.

## 4. Discussion

Energy metabolism is accelerated by physical invasion. REE and REE/body weight on the day after esophageal ESD increased significantly by 12.2% and 14.8%, respectively, compared with those on the day of ESD. The stress factor on POD1 was 1.11, and REE/BEE on the day of ESD was 1.00. In addition, the WBC, neutrophil, and CRP levels were elevated significantly on the day after ESD and were correlated with the change ratio of REE. Therefore, it became clear that the physical invasiveness of esophageal ESD was due to increased energy metabolism and inflammation.

In a previous study of surgical procedures, REE/body weight increased by 31% in 35 Japanese male patients aged 40–76 years on the day after esophagectomy as compared with that in the preoperative period [37]. Furthermore, REE/body weight was 23.3 ± 2.1 kcal/kg/day before esophagectomy, but it was 27.3 ± 3.5 kcal/kg/day on POD7. The REE/body weight on POD7 was significantly higher (by 12%) [12]. Our study showed that the degree of increase in the REE/body weight of esophageal ESD on the next day was approximately half that of esophagectomy, and at the same level of esophagectomy on POD7. The stress factor of esophagectomy was defined as 1.8 on POD3 [38]. Thus, the stress factor of esophageal ESD on the day after surgery was lower than that of esophagectomy on POD3. The present study suggests that esophageal ESD is less invasive than open surgery. 

Our previous study indicated that during the perioperative period of gastric and colorectal ESD, REE/ body weight increased by 7.3% and 6.8%, respectively, on the day after ESD, and the stress factors were 1.07 and 1.06, respectively [13,14]. In this study, the degree of increase in esophageal ESD was higher than that of those procedures, suggesting a higher invasiveness of esophageal ESD in comparison with that of gastric or colorectal ESD.

In addition, the excessive production of inflammatory cytokines in the acute phase response to surgery or infection is known to lead to the activation of leukocytes [39]. On POD1 of gastric and colorectal ESD, the WBC and CRP levels significantly increased [5,40]. As shown in previous prospective studies, WBC and CRP levels were increased on POD1 of colorectal ESD or laparoscopy-assisted colectomy for colorectal cancer [41]. Data obtained from esophageal ESD suggested that inflammation is also related to an increase in REE.

The present study revealed that the total resection area was associated with energy metabolism during the perioperative period of esophageal ESD. One possible reason for this is that oral commensal bacteria affect post-ESD ulcers. In previous studies on gastric ESD, bacteremia was caused by oral commensal bacteria [17], and the degree of total resection area was associated with an increase in REE on POD1 [14]. In the evaluation of serum opsonic activity measured using the chemiluminescence method, the most significant increase was observed on the day after gastric ESD [5]. As with gastric ESD, early stimulation by oral commensal bacteria increases REE in esophageal ESD. Therefore, the total resection area is presumed to be a factor affecting the physical invasiveness of post-esophageal ESD ulcers. Furthermore, post-ESD ulcers with lesions over 6.9 cm^2^ were often approximately half of the circumference of the lumen and had markedly increased REE. Patients with a resection area surpassing half of the lumen require careful postoperative management. 

Previous studies have reported that age, BMI, resection area, operation time, and nutritional status are important factors for predicting prognosis in surgical operations for malignant tumors, including esophageal cancer [40,42,43]. Several studies on age have shown that ESD can be performed safely in older patients [4,44]. Conversely, older patients who underwent gastric ESD with perforations had a longer hospitalization than comparably younger patients [45]. In Japanese patients aged > 80 years, the occurrence of delayed bleeding after gastric ESD was reported to be high [46]. With regard to BMI, the risk of pneumonia increased in the overweight group after gastric ESD [47]; additionally, during perioperative colorectal ESD, a higher BMI (≥ 25 kg/m^2^) tended to increase the risk of hypoxemia [48]. Previous studies reported that a long operation time was a risk factor for the perforation of gastric ESD [49]. On the other hand, our previous study indicated that operation time was not found to affect energy metabolism during the perioperative period of gastric and colorectal ESD [13,14]. In studies that measured sarcopenia, subjects who underwent gastric ESD were at high risk of complications such as pneumonia, hyponatremia, and sepsis [50]. In patients older than 80 years, a high PMI has been shown to be a good prognostic factor associated with long-term survival after gastric ESD [51]. However, a previous report showed that BMI and sarcopenia were not independent prognostic factors for postoperative outcomes after esophagectomy [30]. In the present study, age, BMI, operation time, and sarcopenia did not increase REE. Therefore, esophageal ESD can be performed irrespective of age, BMI, operation time, and sarcopenia.

This study had several limitations. First, it was conducted in a single medical institute. However, we performed esophageal ESD according to standard procedures, sedation, and perioperative management according to the instructions of the JGE Society. Therefore, we obtained similar results to those of other multicenter studies. Second, this was a single-arm study. No control group was established for the evaluation of ESD-associated physical invasiveness, as it would be difficult to set similar conditions for a group of healthy volunteers. Third, measurements were only obtained from our subjects on the day of and the day after esophageal ESD, although previously reported patients with esophagectomy had REE measured on POD3 or POD7. Indeed, most patients who underwent esophageal ESD ingested food again starting on POD2 and were discharged on POD6. However, energy metabolism was considered to return to the same level as that in the preoperative period at the time of discharge.

## 5. Conclusions

Energy metabolism increased on POD1 esophageal ESD and the degree of invasiveness was low compared with that of open surgery. However, esophageal ESD was more invasive than gastric and colorectal ESD. Therefore, our results indicate that the perioperative management of esophageal ESD should be carefully conducted. In addition, careful attention is required when the resection area of the lesions is large, especially when it comprises more than half of the lumen.

## Figures and Tables

**Table 1:** Patient baseline characteristics.

Variables	*n*/Median (Range)
Sex (Male:Female)	67:8
Age (years old)	66 (45–90)
BMI (kg/m^2^)	22.1 (16.2–30.4)
PMI (cm^2^/m^2^)	6.4 (3.2–12.4)
Main tumor location	
Upper thoracic esophagus	6
Middle thoracic esophagus	5
Lower thoracic esophagus	48
Gastroesophageal junction	16
Total resection area (cm^2^)	6.9 (0.5–106.0)
Operation time (minutes)	75 (17–265)
Histologic type	
Squamous cell carcinoma	72
Adenocarcinoma	2
Angioma	1
Complications	
Bleeding	1 (1.3%)
Perforation	0 (0%)
Fever (>38 °C)	4 (5.3%)

Data are presented as number (percentage) or median (range). BMI: body mass index; PMI: psoas muscle mass index.

**Table 2:** Changes in REE, body weight, and BEE.

Measurements	Day of ESD	Day Following ESD
REE (kcal)	1194.7 (608.1–1583.7)	1340.0 * (847.6–2111.3)
Body weight (kg)	59.4 (40.6–86.1)	58.1 * (38.9–86.5)
BEE (kcal)	1235.0 (941.0–1677.0)	1247.1 (983.7–1562.8)

Data are presented as median (range). * *p <* 0.05, vs day of ESD. REE: resting energy expenditure; BEE: basal energy expenditure; ESD: endoscopic submucosal dissection.

**Table 3:** Changes in white blood cells (neutrophils, monocytes, eosinophils, basophils, and lymphocytes), and C-reactive protein.

	Day of ESD	Day Following ESD
White blood cells (/µL)	5660 (2760–10220)	9520 * (3730–21190)
Neutrophils (/µL)	3190 (1310–5640)	7170 * (3010–16690)
Monocytes (/µL)	342 (128–821)	551 * (201–1413)
Eosinophils (/µL)	144 (0–649)	86 * (0–529)
Basophils (/µL)	31 (0–217)	19 * (0–81)
Lymphocytes (/µL)	1740 (679–4560)	1430 * (470–9270)
C-reactive protein (mg/dL)	0.05 (0.02–2.89)	0.76 * (0.04–7.73)

Data are presented as median (range). * *p* < 0.001, vs day of ESD. ESD: endoscopic submucosal dissection.

**Table 4:** Univariate analysis for the factors associated with REE during the perioperative period of ESD.

Variables	*n*	REE during the Perioperative Period		Changes in the Ratio of REE
		Day of ESD	Day Following ESD	
Age (years old)				
<65	33	1250.0 (686.8–1535.0)	1391.0 (1009.1–2111.3)	1.08
≥65	42	1117.0 (608.1–1583.7)	1289.2 (847.6–1671.3)	1.10
BMI (kg/m^2^)				
<25	60	1125.3 (608.1–1532.7)	1293.3 (847.6–2111.3)	1.09
≥25	15	1346.0 (1188.3–1583.7)	1498.0 (859.7–1714.3)	1.08
Total resection area (cm^2^)				
<6.9	37	1188.3 (608.1–1560.7)	1291.3 (847.6–1671.3)	1.07
≥6.9	38	1239.5 (686.8–1583.7)	1397.7 (962.0–2111.3)	1.13 *
Operation time (minutes)				
<75	36	1159.5 (826.0–1583.7)	1286.0 (921.9–2111.3)	1.08
≥75	39	1241.7 (608.1–1537.0)	1397.7 (847.6–1714.3)	1.09
Sarcopenia				
Non-sarcopenia	52	1231.3 (686.8–1583.7)	1344.7 (859.7–2111.3)	1.08
Sarcopenia	23	1111.7 (608.1–1560.7)	1335.7 (847.6–1714.3)	1.12

Data are presented as median (range). * *p* < 0.05, vs. total resection area < 6.9 cm^2^. REE: resting energy expenditure; ESD: endoscopic submucosal dissection; BMI: body mass index.

**Table 5:** Parameter estimates of main effects for changes in REE using generalized linear models.

Variables	Estimates	SE	Odds Ratio	*p*-Value
Age	0.6193	0.4985	1.8577	0.214
BMI	−0.1944	0.6002	0.8233	0.746
Total resection area	0.7855	0.5003	2.1935	0.116
Operation time	0.0576	0.5012	1.0593	0.908
Sarcopenia	0.0690	0.5252	1.0714	0.895

REE: resting energy expenditure; SE: standard error; BMI: body mass index.

## Data Availability

The data presented in this study are available on request from the corresponding author. The data are not publicly available due to privacy and ethical restrictions.
